# Agomir-122-loaded nanoparticles coated with cell membrane of activated fibroblasts to treat frozen shoulder based on homologous targeting

**DOI:** 10.1186/s12951-024-02403-w

**Published:** 2024-04-10

**Authors:** Zhen Peng, Beijie Qi, Zhiwen Luo, Yaying Sun, Xingyu Zhang, Jinrong Lin, Jinhui Pang, Peng Zhang, Zhihu Zhao, Xianwen Wang, Jiwu Chen

**Affiliations:** 1 Department of Sports Medicine, Shanghai General Hospital, Shanghai Jiao Tong University School of Medicine, Shanghai Jiaotong University, 85# Wujin Road, Hongkou District, Shanghai, 200080 China; 2 Department of Orthopedics, Shanghai Pudong Hospital, Fudan University Pudong Medical Center, Shanghai, China; 3 Shanghai Medicine College, Fudan University, Shanghai, 201399 China; 4 Department of Sports Medicine, Huashan Hospital, Fudan University, Shanghai, 200080 China; 5 Department of Orthopaedics, Tianjin Hospital, No. 406, Jiefangnan Road, Hexi District, Tianjin, 300000 China; 6 School of Biomedical Engineering, Research and Engineering Center of Biomedical Materials, Anhui Medical University, Hefei, 230032 China

**Keywords:** Frozen shoulder, microRNA, Fibrosis, Cell membrane, Nanoparticle

## Abstract

As a common musculoskeletal disorder, frozen shoulder is characterized by thickened joint capsule and limited range of motion, affecting 2–5% of the general population and more than 20% of patients with diabetes mellitus. Pathologically, joint capsule fibrosis resulting from fibroblast activation is the key event. The activated fibroblasts are proliferative and contractive, producing excessive collagen. Albeit high prevalence, effective anti-fibrosis modalities, especially fibroblast-targeting therapies, are still lacking. In this study, microRNA-122 was first identified from sequencing data as a potential therapeutic agent to antagonize fibroblast activation. Then, Agomir-122, an analog of microRNA-122, was loaded into poly(lactic-co-glycolic acid) (PLGA) nanoparticles (Agomir-122@NP), a carrier with excellent biocompatibility for the agent delivery. Moreover, relying on the homologous targeting effect, we coated Agomir-122@NP with the cell membrane derived from activated fibroblasts (Agomir-122@MNP), with an attempt to inhibit the proliferation, contraction, and collagen production of abnormally activated fibroblasts. After confirming the targeting effect of Agomir-122@MNP on activated fibroblasts in vitro, we proved that Agomir-122@MNP effectively curtailed fibroblasts activation, ameliorated joint capsule fibrosis, and restored range of motion in mouse models both prophylactically and therapeutically. Overall, an effective targeted delivery method was developed with promising translational value against frozen shoulder.

**Graphical Abstract:**

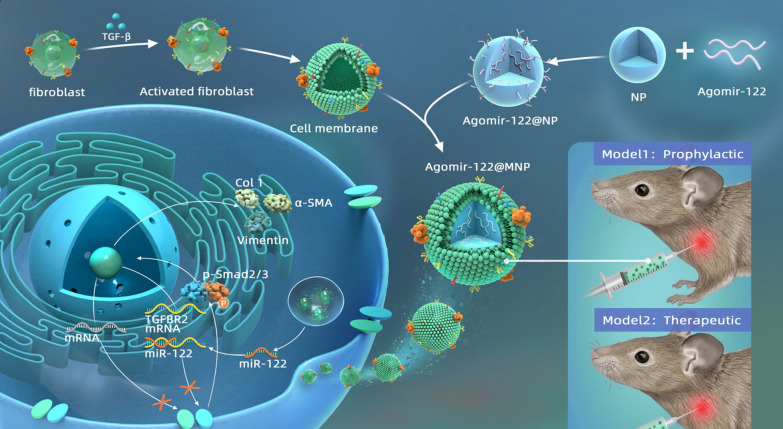

**Supplementary Information:**

The online version contains supplementary material available at 10.1186/s12951-024-02403-w.

## Introduction

Frozen shoulder is a common disorder in orthopedics and sports medicine. Characterized by loss of range of motion (ROM) and pain, the disease affects 2–5% of the general population and can last for years, inducing disability and adding physical and mental burden. In patients with systemic disorders such as diabetes mellitus, the incidence of frozen shoulder can reach 20% [1]. Joint capsule fibrosis, thickening, and stiffening are the classical manifestations of frozen shoulder. Microscopically, these changes are driven by activated fibroblasts, which have higher activity, proliferation, and contraction ability [2]. However, despite a clear understanding of the pathologic manifestation, the pathological mechanisms underlying the activation of fibroblasts remain unclear. How to treat/prevent frozen shoulder is still a challenge. Popular modalities include the elimination of inflammation by oral or injectable medication to impede the fibrotic process and physical exercises to soften the stiffened joint capsule, but it should be noted that only some patients respond to these procedures. In one-third of cases, frozen shoulder cannot recover and becomes refractory [3]. At this stage, the only effective method is surgery. Therefore, there is an unmet need for novel therapeutic agents.

Contrary to the rich categories of anti-inflammatory agents, such as steroids, hyaluronic acid, and bee venom, commercially available agents with direct anti-fibrotic effects are still lacking in the clinic. Fitzpatrick et al. tested the effect of collagenase injection for patients with frozen shoulder, but the results were disappointing and did not support the additional benefit of this method against control [4]. A better understanding of pathological mechanisms is necessary.

At present, fibroblast activation is deemed a central phenomenon in this disease. Physiologically, fibroblasts are a set of nonhematopoietic structural cells that participate in the maintenance of tissue architecture, development, and repair after injury [5]. On the other hand, once aberrantly activated, fibroblasts become more migrative, proliferative, and contractive and are primarily responsible for the synthesis of extracellular matrix and the remodeling of tissue [6], resulting in excessive collagen synthesis in the shoulder capsule and finally leading to progressively limited passive ROM [7].

To date, multiple mechanisms, including but not limited to insulin resistance, chronic hypoxia, and endotoxemia, have been proposed [8]. Based on our previous findings, routine blood-based markers can be prognostic for postoperative frozen shoulder, and preoperative serum has profibrotic potential [9]. These clues pinpoint the importance of systemic conditions and the onset of frozen shoulder.

Countless materials, including metabolites, lipids, and proteins, are enriched in the circulation. In recent years, the importance of circulating exosomes has been emphasized. Exosomes are extracellular vesicles with a size of 30–150 nm [10]. Its biological function relies on the cargo, which contains proteins, nucleic acids, etc. [11]. Our exosomal miRNA sequencing found that circulating exosomes from patients with frozen shoulder had significantly higher levels of specific miRNAs and had a protective effect against fibrosis, which might be a self-protecting effect against fibrosis. Enlightened by this phenomenon, miRNA supplementation can be a good method for inhibiting the pro-fibrotic phenotype of fibroblasts.

To selectively elevate the level of miRNAs in activated fibroblasts, agent loading and targeted delivery systems are needed. Advances in nanotechnology have provided a variety of strategies to achieve this goal, among which PLGA nanoparticles have good biocompatibility and are hence perfect candidates for miRNA loading [12, 13]. Moreover, relying on the affinity between homologous cells, coating nanoparticles with specific cell membranes can enhance delivery efficiency into target cells and escape the immune response from macrophages. For example, Fe_3_O_4_ nanocubes were cloacked with cancer cell membrane to improve the targeting property of toxic nanomaterials into Hela cells [14]. Qu et al. coated multiple myeloma cell membrane onto bortezomib-loaded nanoparticles for boosting bone marrow homing via homologous targeting [15]. Wu et al. wrapped nanoprobes with melanoma cell membrane to achieve better drug loading efficiency, targeting capacity, and anti-tumor efficiency [16].

**Scheme 1:**
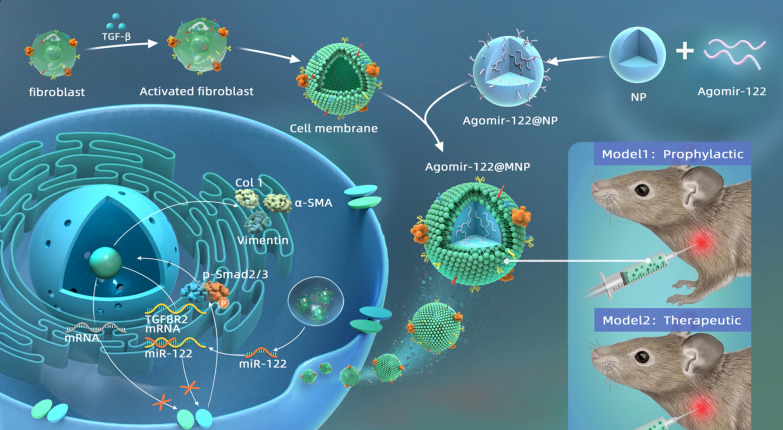
Scheme of cell membrane-coated nanoparticles loaded with Agomir-122 for the treatment of frozen shoulder. The coating of cell membrane derived from activated fibroblasts enables Agomir-122 loaded nanoparticles to target and enter activated rather than inactivated fibroblasts, by which this strategy antagonize fibrotic process. The experiments in vivo support its effectiveness in preventing and treating frozen shoulder

Therefore, in this work, we first analyzed our previous miRNA sequencing data (GSE182896), and selected miRNA-122 as the candidate because of its anti-fibrotic potential. We then selected Agomir-122, the analog of miRNA-122 with better stability in vivo, for subsequent studies. Agomir-122 was loaded into PLGA nanoparticles with the aim of retarding the abnormal activation of fibroblasts. Next, with the aid of homologous targeting effect, we harvested the cell membrane of activated fibroblasts and coated the membrane onto the miRNA-loaded nanoparticles. Through this technique, we constructed a targeting delivery system with an attempt to treat frozen shoulder. Both preventative and therapeutic effect was tested in a mouse model (Scheme 1).

## Results and discussion

**Fig. 1:**
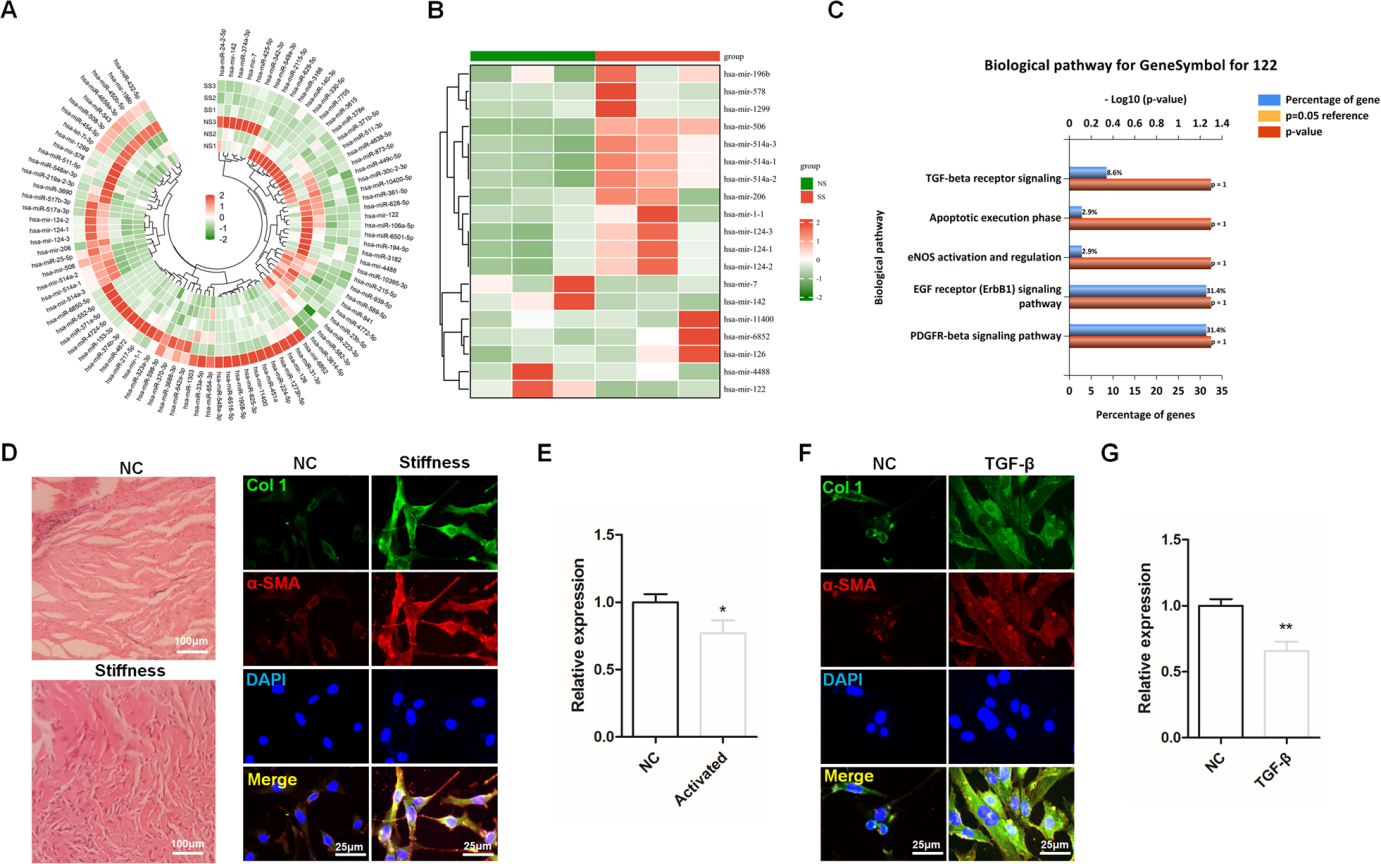
The level of miR-122 in different conditions. **A** Top 100 differentially expressed exosomal miRNAs between patients with frozen shoulder and those without (GSE182896). **B** Heatmap of all 19 differentially expressed miRNAs with statistical significance. **C** Top biological pathways miR-122 enrolled in. **D** HE staining of joint capsule samples of patients with or without frozen shoulder, and immunofluorescent staining of fibroblasts derived from samples, respectively. The NC group consisted of joint capsule samples from patients without frozen shoulder, and the stiffness group consisted of patients with frozen shoulder (n = 3). **E** The levels of miR-122 in CDFs extracted from samples with and without frozen shoulder. **F** Immunofluorescent staining of fibroblasts from samples without frozen shoulder challenged by TGF-β. **G** The level of miR-122 in fibroblasts without frozen shoulder challenged by TGF-β. Scale bar: 25 μm. *p < 0.05. **p < 0.01. *NC* Negative control

Previously, we conducted exosomal miRNA sequencing (top 100 differentially expressed miRNAs shown in Fig. 1A) and found that four exosomal miRNAs, i.e., miR-7-1-3p, miR-4488, miR-142-3p, and miR-122-5p, were significantly up-regulated in those with frozen shoulder compared with those without frozen shoulder (Fig. 1B), and the counts of miR-122-5p per million reads far exceeded those of the other three (detailed information in GSE182896). Biological pathway analysis indicated that miR-122 was involved in the regulation of the well-recognized pro-fibrotic signal, i.e., the TGF-β pathway (Fig. 1C), and available evidence supported its role in curtailing TGF-β receptor 2 expression [17]. Therefore, this miRNA was selected as the potential candidate to relieve fibrosis.

The level of miR-122 in CDFs (capsule-derived fibroblasts) under different conditions was then measured. To guarantee the quality of the CDFs we harvested, the joint capsule samples were examined first. In accordance with previous reports [18, 19], the fibrils of the joint capsule from patients with frozen shoulder were denser (Fig. 1D), indicating stiffened shoulder and joint capsule fibrosis. Next, CDFs from these joint capsule samples were confirmed to have increased expression of α-SMA and Col 1, confirming the activated status of CDFs (Fig. 1D). On the other hand, contrary to the expression profile in exosomes, miR-122 was significantly down-regulated in these activated CDFs (Fig. 1E).

When CDFs from patients without frozen shoulder were challenged with TGF-β, both α-SMA and Col 1 were up-regulated (Fig. 1F), while miR-122 (Fig. 1G) was significantly down-regulated, which was in line with other studies [20, 21]. This data suggested that, for CDFs from patients without frozen shoulder, TGF-β could induce activation, mimicking the profile of primary CDFs from those with.

Therefore, in the following experiments, we first expanded CDFs from patients without frozen shoulder to passages 6–7, a status maintaining fibroblast phenotype [22]. Then, we used TGF-β to artificially activate cells to harvest cell membrane. In this manner, large-scale production of the cell membrane of activated CDFs was achieved, providing feasibility for translational medicine.

Agomir refers to the double-stranded miRNA mimic with chemical modification, which is widely used for up-regulating miRNA in cells. By introducing Agomir-122 into activated fibroblasts in a targeted manner, anti-fibrotic therapy against frozen shoulder could be achieved. To achieve this, we first obtained poly PLGA nanoparticles (NP). The loading efficacy of Agomir-122 into NP (Agomir-122@NP) was 0.25%.

**Fig. 2:**
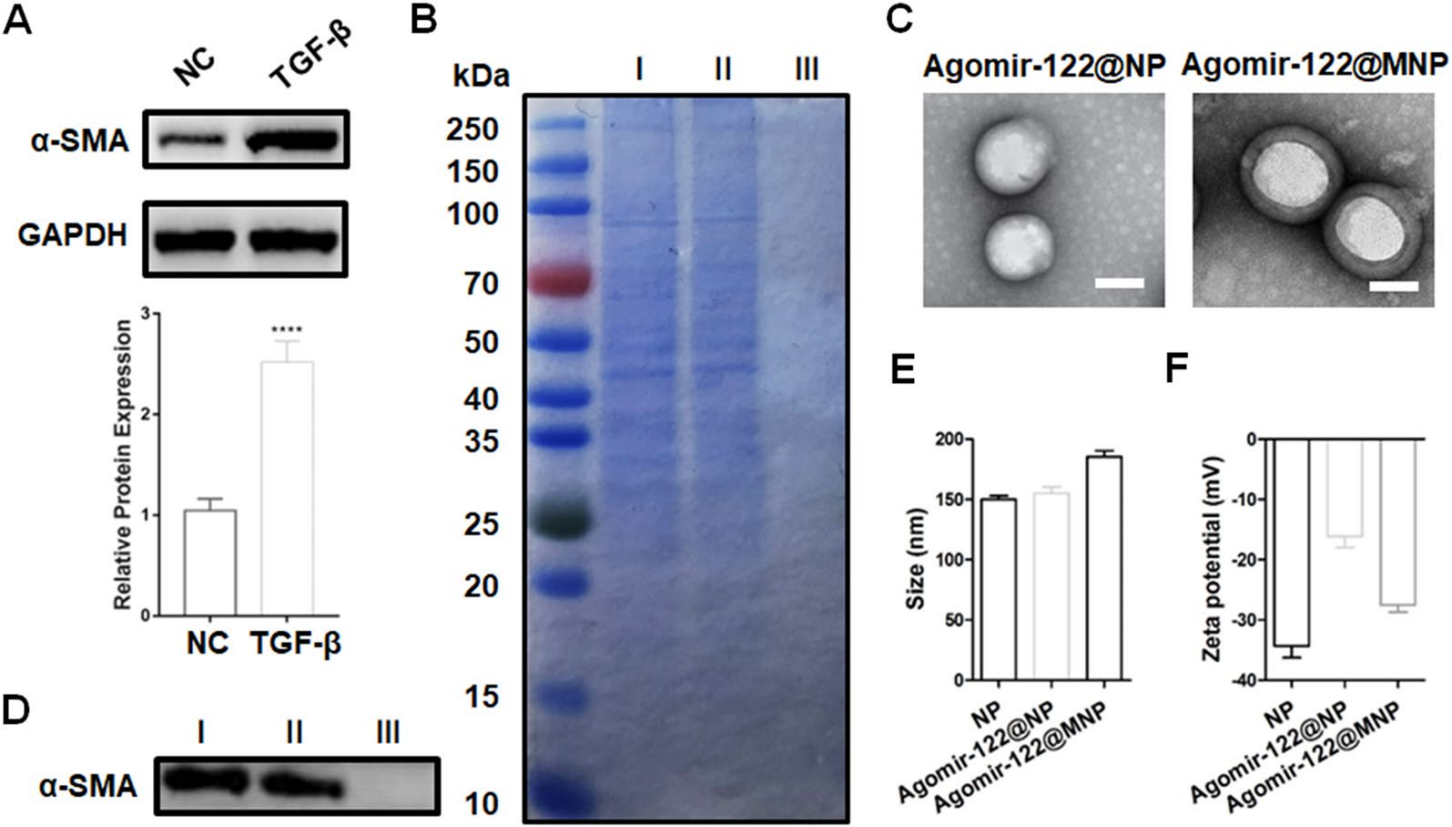
Characterization of Agomir-122 loaded nanoparticles wrapped with cell membrane of activated CDFs. **A** The level of α-SMA in CDFs with TGF-β stimulation, determined by western blot, and quantification (n = 3). **B** SDS-PAGE analysis of CDF, Agomir-122@MNP, and Agomir-122@NP. **C** Morphology of Agomir-122@MNP and Agomir-122@NP, observed by STM. Scale bar: 100 nm. **D** The detection of α-SMA by western blot regarding the obtained cell membrane (I), Agomir-122@MNP (II), and Agomir-122@NP (III). **E** Diameters of NP, Agomir-122@NP, and Agomir-1222@MNP (n = 3). **F** Zeta potentials of NP, Agomir-1222@NP, and Agomir-122@MNP (n = 3). ****p < 0.0001

The cell membrane of TGF-β-activated CDFs was collected and proved to have extreme α-SMA expression (Fig. 2A). After Agomir-122@NP was coated with this membrane (Agomir-122@MNP), sodium dodecyl sulfate polyacrylamide gel electrophoresis (SDS-PAGE) confirmed the existence of proteins on Agomir-122@MNP, as detected in CDFs (Fig. 2B). Transmission electron microscopy (TEM) images illustrated that, Agomir-122@NP was successfully prepared in a round shape, while Agomir-122@MNP had a spherical core and outer membrane layer (Fig. 2C). WB proved that, both the cell membrane and Agomir-122@MNP, but not Agomir-122@NP, had α-SMA expression (Fig. 2D). Finally, average size and zeta potential were measured. The average diameter of the NP was 150.0 ± 2.5 nm, that of Agomir-122@NP was approximately 154.9 ± 4.5 nm, and that of the Agomir-122@MNP was around 185.4 ± 4.1 nm (Fig. 2E). The charge of the NP was − 34.3 ± 1.6 mV, that of Agomir-122@NP was − 16.1 ± 1.5 mV, and that of Agomir-122@MNP declined to − 27.5 ± 1.0 mV (Fig. 2F). These findings together proved a successful preparation of Agomir-122@MNP.

**Fig. 3:**
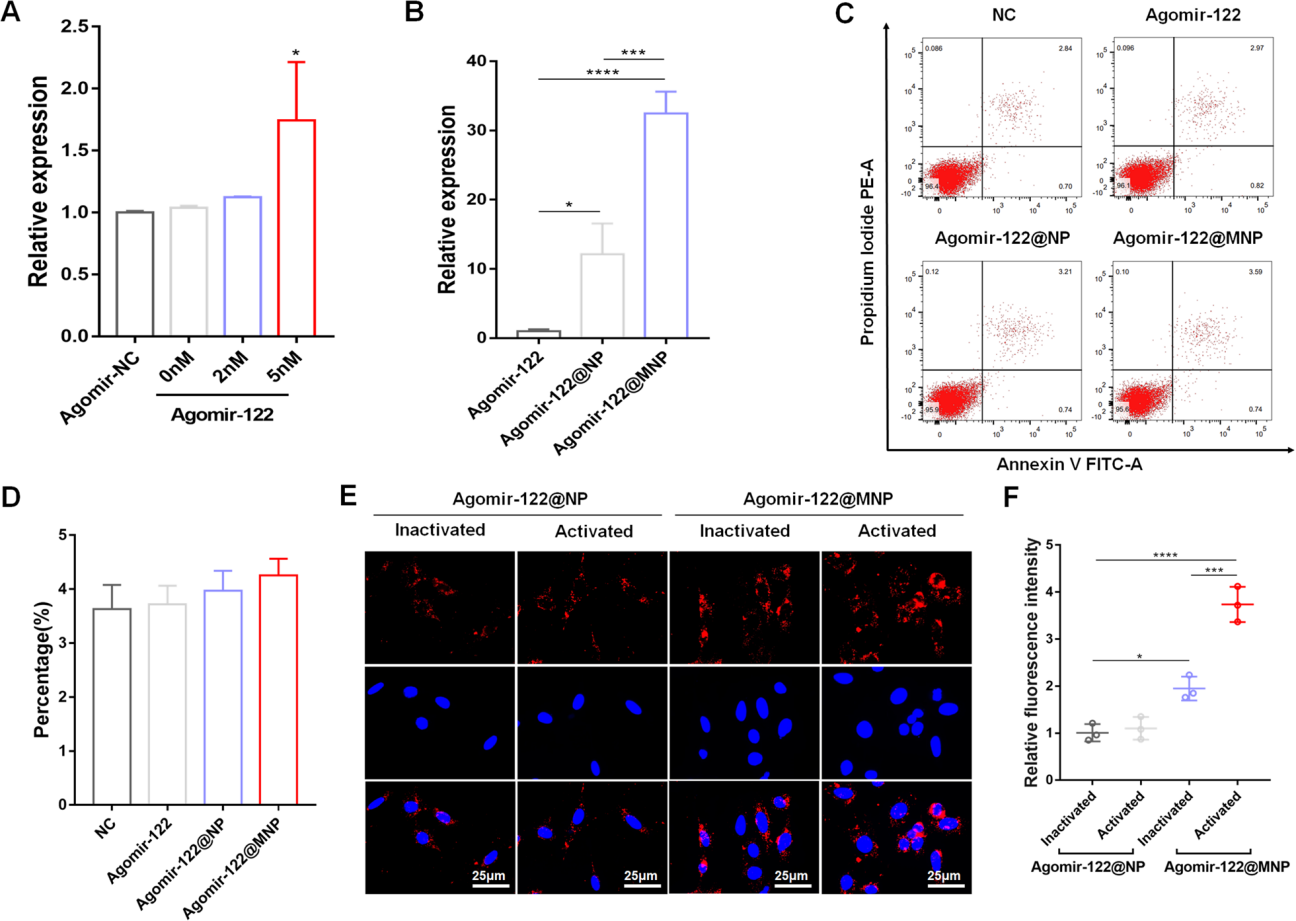
Biological characteristics of Agomir-122@MNP. **A** Determining a concentration of Agomir-122 effective in up-regulating intra-cellular level of miR-122. **B** The influence of Agomir-122, Agomir-122@NP and Agomir-122@MNP on the expression of miR-122 in CDFs. **C** The biosafety of different agents on CDFs, as measured by apoptosis flow cytometry. **D** Quantification of percentage of apoptosis. **E** The uptake of Agomir-122@NP or Agomir-122@MNP (DiR-labeled) by inactivated and activated CDFs. **F** Quantification of uptake of agents by cells. (n = 3). Scale bar: 25 μm. *p < 0.05; ***p < 0.001; ****p < 0.0001

Next, we tested the biocompatibility and targeting efficiency of Agomir-122@MNP. First, to determine a concentration of Agomir-122 capable of significantly elevating miR-122 expression in activated CDFs, gradient stimulation was conducted. The concentration of 5 nM could significantly up-regulate the intra-cellular level of miR-122 (Fig. 3A). Based on loading efficiency, the amount of Agomir-122@NP required to reach this concentration was obtained, and we found that and the up-regulation of miR-122 was enhanced by Agomir-122@NP and Agomir-122@MNP (Fig. 3B). Moreover, this interference did not induce significant apoptosis of activated CDFs, as indicated by percentage of cells were stained Propidium Iodide (Fig. 3C and quantified in D). The targeting efficiency was also tested. Dil-labeled Agomir-122@NP could be internalized into both inactivated CDFs (derived from patients without frozen shoulder) and activated CDFs (from patients with frozen shoulder) without significant difference, but Dil-labeled Agomir-122@MNP preferably entered activated CDFs than inactivated cells (Fig. 3E and quantified in F).

**Fig. 4:**
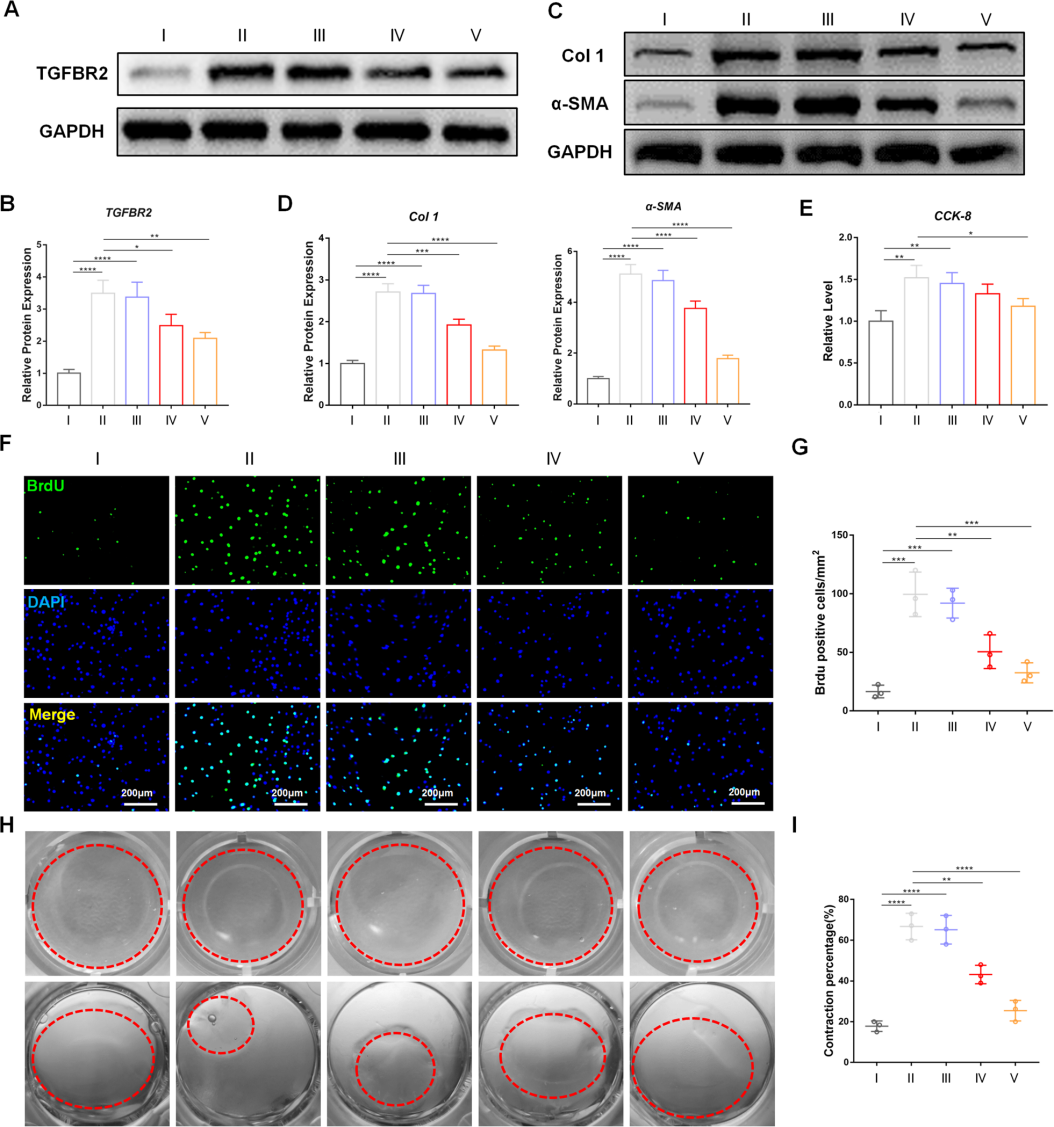
The anti-fibrotic effect of Agomir-122@MNP on activated CDFs. **A** TGFBR2 expression of inactivated CDFs and activated CDFs with different interventions, as indicated by western blot. **B** Quantification of TGFBR2 expression. **C** Col 1 and α-SMA expression of inactivated CDFs and activated CDFs with different interventions, as indicated by western blot. **D** Quantification of Col 1 and α-SMA expression. **E** Cell activity of inactivated CDFs and activated CDFs with different interventions, as indicated by CCK-8 assay. **F** Cell viability of inactivated CDFs and activated CDFs with different interventions, as indicated by BrdU staining (green). **G** Quantification of BrdU staining. **H** Cell contraction ability of inactivated CDFs and activated CDFs with different interventions, as indicated by collagen gel contraction assay (red circle indicated the collagen gel). **I** Quantification of collagen gel area. Scale bar: 200 μm. (n = 3). *p < 0.05; **p < 0.01; ***p < 0.001; ****p < 0.0001. I: Unactivated CDFs, II: Activated CDFs, III: Agomir-122 + activated CDFs, IV:Agomir-122@NP + activated CDFs, V: Agomir-122@MNP + activated CDFs

The anti-fibrotic effect of Agomir-122@MNP on activated CDFs was then tested. Firstly, the expression of TGFBR2 was examined. Compared to inactivated cells, activated cells had significantly higher level of TGFBR2, but Agomir-122, Agomir-122@NP, and Agomir-122@MNP gradually decreased this expression (Fig. 4A and quantified in B). It should be noted that Agomir-122 alone failed to induce significant decline of TGFBR2 in vitro. This phenomenon might be attributed to the fact that, although this agent could significantly induce miR-122 up-regulation, the fold-change was still limited, therefore the biological effect was not remarkable. Besides, the overall up-regulation of miR-122 could be a consequence of particularly high miR-122 level within only a small proportion of cells, which had limited impact on the expression of TGFBR2 as a whole.

For canonical TGF-β signaling pathway, TGF-β binds with TGFBR2 to recruit TGFBR1 and phosphorylate Smad2/3 (p-Smad2/3) in the cytoplasm [23], and the latter is then transported into nuclei to initiate pro-fibrotic process, which leads to extreme expression of Col and α-SMA [24]. Therefore, the level of p-Smad2/3 was determined. Immunofluorescent staining showed that, compared to inactivated cells, p-Smad2/3 not only ascended significantly in activated cells, but also accumulated in nuclei. On the other hand, the level of p-Smad2/3 was significantly inhibited by Agomir-122@NP, and was further retarded by Agomir-122@MNP (Additional file 1: Figure S1). This observation reflected that the Smad signaling was suppressed by Agomir-122@MNP.

The level of Col 1 and α-SMA were then quantified. In accordance with the expression of TGFBR2, both markers elevated significantly in activated CDFs, and decreased in a gradient manner following Agomir-122, Agomir-122@NP, and Agomir-122@MNP stimulation (Fig. 4C and quantified in D). In fibrotic tissue, the hallmarks are the activation of CDFs, which is characterized by enhanced cell viability, proliferation, and contractile ability [9]. By conducting CCK-8 assay (Fig. 4E), BrdU staining (Fig. 4F and G), and collagen contraction test (Fig. 4H and I), we proved that the activation of fibroblasts is effectively retarded. All these data supported the anti-fibrotic role of Agomir-122@MNP.

Usually, frozen shoulder can be divided into inflammatory stage, fibrotic stage, and restoration stage [7]. The aforementioned experiments have confirmed the role of Agomir-122@MNP in inhibiting the already-existed activation of CDFs derived from fibrotic joint capsule, but whether this nanoparticle system can prevent CDFs from being activated is not clear. For patients with frozen shoulder which is about to enter fibrotic stage or have risk factors of developing frozen shoulder, it is necessary to give prophylactic interventions. Therefore, the impact of Agomir-122@MNP on CDFs that are being activated should also be examined.

To establish a model in vitro, CDFs from patients without frozen shoulder were used. By simultaneously treating cells with TGF-β and Agomir-122@MNP, the effect of this agent on the ongoing fibrotic process was measured. Similarly, we noticed that TGF-β remarkably promoted cell proliferation, activity, and contractile ability were all mitigated by Agomir-122@MNP (Additional file 1: Figure S2). These outcomes confirmed that Agomir-122@MNP could not only inhibit the activated status of CDFs, but also deter CDFs from being activated.

To make the aforementioned results more robust, the therapeutic effect of this agent was also tested in vivo in a mouse model. Immobilized shoulder of rodents is well-acknowledged as a good model to investigate joint capsule fibrosis, as this model shares the similar characteristics to clinical findings, such as thickening of shoulder joint capsule, infiltration of inflammatory cells, and ROM deficiency [18, 22].

Prior to delivering the agent into mouse shoulder joint, we harvested cell membrane from NIH3T3 mouse fibroblasts (M^m^) activated by TGF-β (Additional file 1: Figure S3), and manufactured Agomir-122@M^m^NP for experiments in murine cells and joints (Additional file 1: Figure S4). For NIH3T3 cells treated with TGF-β, Agomir-122 of 20 nM could significantly up-regulate the expression of miR-122 in vitro (Additional file 1: Figure S5A), which was further strengthened by Agomir-122@M^m^NP (Additional file 1: Figure S5B). Neither Agomir-122@NP nor Agomir-122@M^m^NP was cytotoxic (Additional file 1: Figure S6). What is more, Agomir-122@M^m^NP was selectively internalized in NIH3T3 fibroblasts activated by TGF-β (Figure S7, Supporting information), and escape the engulfment of RAW264.7 macrophages (Additional file 1: Figure S8). The anti-fibrotic effect of Agomir-122@M^m^NP was then measured in vitro. Not surprisingly, cell proliferation, viability, and contraction ability were suppressed (Additional file 1: Figure S9). These data supported the application of this agent in vivo. Meanwhile, in order to exclude whether this antifibrotic effect is produced by the delivery vehicle itself. We performed control experiments on activated NIH3T3 cells and found that Agomir-NC, NP, and MNP did not produce antifibrotic effects by themselves (Additional file 1: Figure S10).

Since frozen shoulder is a gradual process, early intervention is key to preventing fibrosis. On this basis, a prophylactic model of mouse frozen shoulder was used. Tracing analysis showed that, following injection, the signal could be detected within the joint for 1 week (Additional file 1: Figure S11).

**Fig. 5:**
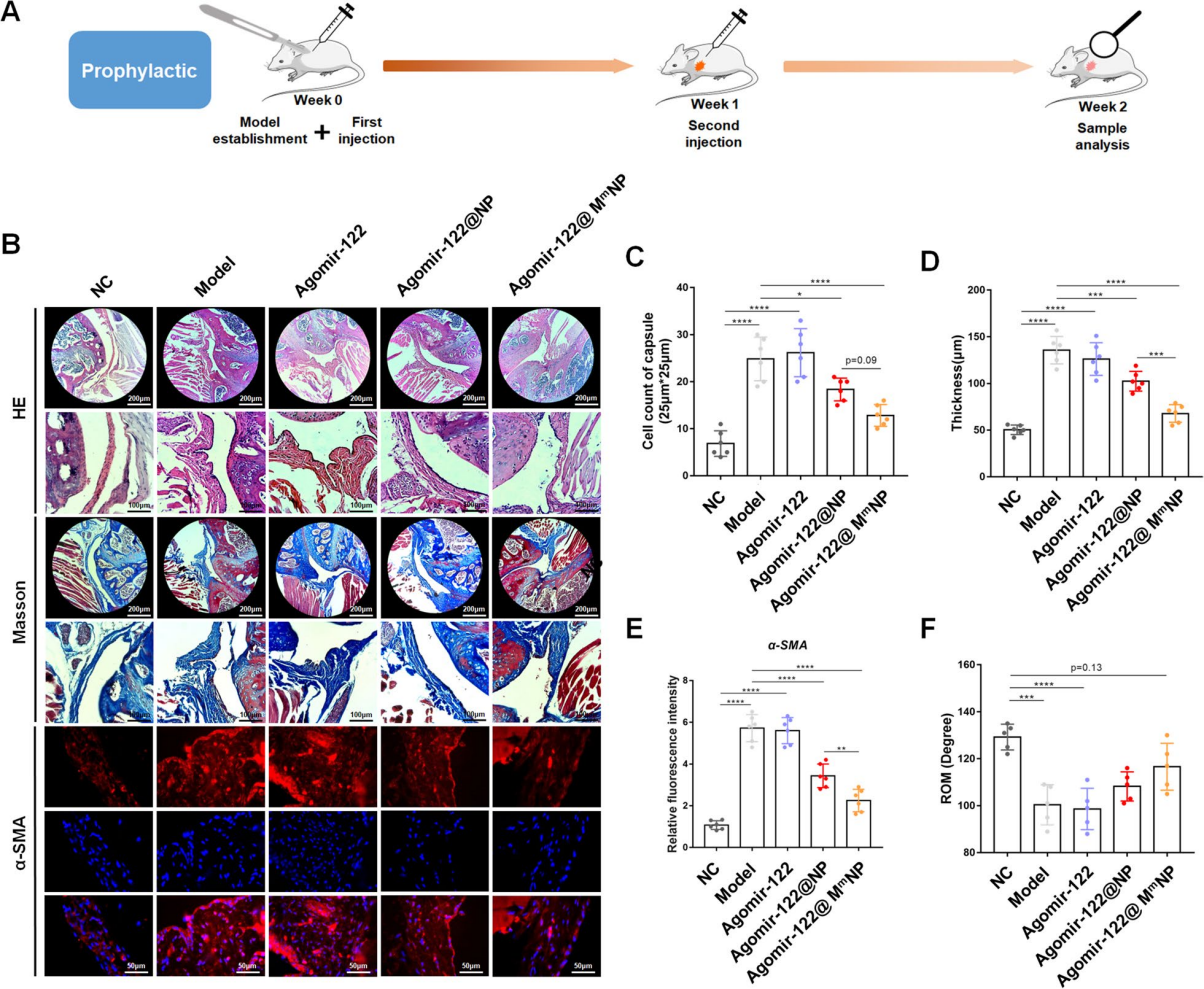
Prophylactic effect of Agomir-122@M^m^NP against frozen shoulder in a mouse model. **A** Schematic chart of model establishment, intervention, as well as sample harvest and observation. **B** Representative histological image of mouse shoulder joint capsule with different interventions, as illustrated by HE, Masson, and α-SMA staining (α-SMA in red and DAPI in blue). **C** Quantification of cell counts in joint capsule of different groups. **D** Quantification of joint capsule thickness of different groups. **E** α-SMA expression in joint capsule of different groups. **F** Passive ROM of the index shoulder of different groups. Scale bar: 200 or 100 μm. (n = 6). *p < 0.05; ***p < 0.001; ****p < 0.0001

Immediately after shoulder immobilization, an injection of Agomir-122@M^m^NP was performed. One week after surgery, the other injection was administered. Two weeks after model establishment, capsule samples were harvested for analysis (Fig. 5A). HE staining showed that, following shoulder immobilization, excessive cell infiltration was detected in shoulder capsule samples of group Model with increased thickness (Fig. 5B–D). However, both cell infiltration and capsule thickening were relieved by Agomir-122@NP intervention, and further alleviated by Agomir-122@M^m^NP (Fig. 5B–D). This restoration was also observed on Masson staining, which identified less collagen fibrils in joint capsule samples with Agomir-122@NP or Agomir-122@M^m^NP intervention compared to group Model (Fig. 5B). Moreover, immunofluorescent staining noticed remarkable accumulation of α-SMA in the immobilized shoulder capsules than that of group NC, but this accumulation was significantly attenuated in group Agomir-122@NP and group Agomir-122@M^m^NP (Fig. 5B and quantified in E). Accordingly, the ROM of Agomir-122@NP group and Agomir-122@M^m^NP group increased gradually than that of group Model (Fig. 5F). In sum, by quantifying cell infiltration, capsule thickness, expression of α-SMA, and ROM, we found both Agomir-122@NP and Agomir-122@M^m^NP exerted anti-fibrotic potential in vivo, and Agomir-122@M^m^NP owned better anti-fibrotic effect.

In clinical practice, it is common to see patients who complain about shoulders with extreme deficiency of ROM, reflecting a status of existing fibrosis. Under this circumstance, how to inhibit the already-fibrotic status is the major concern. Therefore, we also tested the therapeutic potential of Agomir-122@M^m^NP against existed frozen shoulder.

In this model, the first injection was given 1 week after the model establishment, when the shoulder capsule was already thickened and fibrotic. Three weeks after model establishment, capsule samples were harvested (Additional file 1: Figure S12A). By conducting similar analyses, we proved that the Agomir-122@M^m^NP could inhibit cell infiltration, capsule thickening, and α-SMA deposition and restore the ROM of the stiffened shoulder joints, thereby exerting a therapeutic effect against existing frozen shoulder (Additional file 1: Figure S12B–S12F). Finally, biosafety evaluation was conducted. Both major organs (Additional file 1: Figure S13), suggesting that Agomir-122@M^m^NP may not have systemic toxicity.

## Conclusion

In this research, we obtained a cell membrane-coated nanoparticle system loaded with agomir-122 to target activated fibroblasts for the suppression of fibrosis. In vitro, we noticed that agomir-122@MNPs were preferably taken up by activated fibroblasts and escaped engulfment by macrophages. Moreover, this agent significantly inhibited the activity, proliferation, and contraction ability of activated fibroblasts. The anti-fibrogenesis potential of this agent was further tested in vivo. By using prophylactic and therapeutic models, we confirmed that this nanoparticle system could not only curtail the formation of joint fibrosis but also ameliorate existing joint fibrosis. Moreover, intra-articular delivery of this agent did not influence the normal joint structure or have obvious systemic safety concerns.

There are several advantages of our Agomir-122@MNP delivery system. First, sequencing provided a comprehensive view of what happened in the circulating exosomal miRNAs in patients with versus without frozen shoulder, providing rationality for the selection of miR-122. Second, coating cell membranes from activated fibroblasts guaranteed the targeting efficacy of our nanoparticles, which facilitated the delivery of agomir-122 into specific cells. Third, fibrosis is a common manifestation of many disorders. This strategy has the potential to be applied for other fibrotic diseases.

## Methods

This study was approved by the institutional ethical committee of Shanghai General Hospital, Shanghai Jiaotong University (2022KY014). This study was organized following the Declaration of Helsinki. All animal experiments were approved by the local Institutional Animal Care and Use Committee. All procedures were conducted according to the Guide for the Care and Use of Laboratory Animals.

### Nanoparticle preparation

Agomirs was obtained from Ribobio (Guangzhou, China). The sequence of Agomir-122 was 5ʹ-UGGAGUGUGACAAUGGUGUUUG-3ʹ, while that of Agomir-NC was 5ʹ-UCACAACCUCCUAGAAAGAGUAGA-3ʹ. For preparation of Agomir-122 loaded nanoparticles, an aqueous solution of Agomir-122 was added to PLGA (50 mg) dissolved in dichloromethane (2.5 ml) and emulsified by sonication. The obtained colostrum was added to 10 ml of a 1% sodium cholate solution and emulsified by sonication again. The obtained emulsion was then added to 60 ml of 1% sodium cholate solution and agitated for 4 h. The solution was washed 3 times with PBS, and the Agomir-122 nanoparticle (Agomir-122@NP) was obtained.

To measure loading efficiency, Agomir-122@NP was added into 200 DCM for dissolution. Then same volume of TE buffer was added to the NPs and shook for 3 h at 1000 rpm. The NPs were centrifuged at 4 ℃ for 10 min at 15,000 rpm. The absorbance of the supernatant was measured at 260 nm via Nanodrop.

Fibroblasts of ~ 80% conflueny were harvested in IB-1 solution and homogenized thoroughly [25]. The mixture was centrifuged at 4 ℃ (2000 *g*, 10 min) for 3 times to remove nuclei, and membrane was further separated via centrifugation at 4 ℃ (21,000 *g*, 30 min). Finally, Agomir-122 nanoparticle and cell membranes was mixed and prepared by extrusion through filters with 200 nm pore size using Avanti Mini-Extruder (Avanti Polar Lipids).

### Nanoparticles characterization

Zeta potential measurements and size distribution were done by using Nicomp 380ZLS (Nicomp, USA). The surface appearance and inner structure of the nanoparticles were examined by transmission electron microscopy (TEM) Hitachi H-7650 (Hitachi, Tokyo, Japan).

SDS-PAGE protein analysis was conducted to detect the existence of cell membrane proteins on nanoparticles, sample of each group were added into SDS buffer, heated at 70 ℃ for 5 min and placed on a shaker for 90 min. 15 μg of each sample was added into each well of the SDS–polyacrylamide gel and run at 120 V for 120 min. Then the gel was stained with Commassie brilliant blue and washed with decolorization solution until clear [26].

### Cell culture

Two kinds of fibroblasts, CDFs and NIH3T3s, and murine macrophage Raw264.7 cells were used. NIH3T3s and Raw264.7 cells were purchased from Shanghai Cell Bank, while CDFs were harvested from fresh capsular samples from patients with or without frozen shoulder according to the published procedure [9]. Cells were cultured in high-glucose DMEM medium (Thermo Fisher Scientific) plus 10% Certified Fetal Bovine Serum, FBS (VivaCell, Shanghai, China, C04001-500) with 1% penicillin/streptomycin in a humidified incubator with 5% CO_2_ atmosphere at 37 ℃.

### Stem-loop quantitative polymerase chain reaction (qPCR)

Stem-loop qPCR (Taq-Man) was performed to measure the expression of miR-122 with U6 as reference based on the previous protocol [27]. miRNA promoter forward: 5ʹ-CGGGGTACCGTGAGCCCTCCTTGTTAT-3ʹ reverse: 5ʹ-CCGCTCGAGTTGGGTGTCAGGGTAGTC-3ʹ, Comparative Ct method was conducted to evaluate the value of miR-122 (n = 3). All values were normalized by group NC.

### BrdU staining

The proliferation ability of CDFs and NIH3T3 was assessed by a 5-Bromo-2ʹ-deoxyuridine (BrdU) incorporation assay kit (Cell Signaling Technology, MA, USA) according to the manufacturer’s instructions. Briefly, CDFs and NIH3T3 were seeded into a 6-well petri dish with 10^6^ cells/well and then cells were incubated with different interventions for 24 h. Next, BrdU solution was added to each well for another 12 h. After fixed and washed with PBS, cells were incubated with mouse anti-BrdU-primary antibody for 1 h. Finally, DAPI was applied to counterstain the nucleus. Cell proliferation was evaluated using the average number of BrdU positive cells/number of DAPI per view.

### Collagen contraction test

The ability of collagen contraction was measured by collagen contraction test. CDFs and NIH3T3 with the density of 2.0 × 10^5^ cells/ml were seeded in 24-well plates with 1 ml growth medium. Collagen was detached from the plates by 100 μl-sterile pipette tips. Next, the plates were put into an incubator with 37 ℃ and 5% CO_2_. Finally, the plates were observed and pictured by screening at 0 h and 24 h. The collagen area was measured by ImageJ software (NIH, Bethesda, MD, USA) to assess the relative contraction ratio comparing to NC group.

### CCK-8 assay

The viabilities of fibroblasts was measured by cell counting kit-8 (CCK-8, Beyotime Biotechnology, Shanghai, China) assay [28]. Cells were seeded into 96-well dish with the density of 10^3^ cells/well and treated with different interventions for 24 h. CCK-8 solution of 10 μl was added into each well and cells were cultured for 2 h. The absorbance (450 nm) was measured by Microplate Reader (Bio-Rad, Hercules, CA, USA). Cell viabilities were evaluated by average absorbance of different interventions/absorbance of control group.

### Western blot

Proteins were extracted from cells using RIPA buffer (R0010; Solarbio, China) which contained Phenyl methane sulfonyl fluoride (PMSF; Solarbio, China). BCA Protein Assay Kit (Beyotime Biotechnology, China) was utilized to measure the concentration of protein. Protein samples (10 μg each) were added and separated by 10% SDS-PAGE, and transferred to nitrocellulose membranes. After blocking for 1 h, blots were incubated with primary antibodies: α-SMA (Affinity, AF1032), p-Smad2/3 (Affinity, AF3367), Col 1 (Affinity, AF7001), and TGFBR2 (Abcam, ab269279) at 4 ℃ overnight. Afterward, the blots were incubated with secondary antibody at 1:2000 for 1 h at RT. Protein bands were observed and pictured using SYNGENE imaging system (Cambridge, UK), and analyzed by ImageJ (NIH, Bethesda, MD, USA). Three protein samples per group were used for calculation (n = 3/group).

### Establishment of animal model

C57/6 J mice of 10 weeks purchased and used in the current study. The frozen shoulder model was established by immobilization procedure [9]. Inrta-articular injection was conducted anteriorly via sub-acromial portal. For prophylactic model, the first injection was performed immediately after immobilization. One week later, the second injection was performed. Two weeks after immobilization, mice were euthanized by overdose CO_2_ to harvest the shoulders. For therapeutic model, the first injection was conducted 1 week after immobilization and the second injection was conducted at the end of 2 weeks after model establishment. Mice were sacrificed at the end of the 3rd week. Immediately after sample collection, passive range of motion (ROM) was measured with reference to the manner by Oki et al. [19]

### Histological staining

The embedded shoulder joints were cut into 5 μm thick sections using a paraffin slicing machine (LEICA RM2235). Sections were then dewaxed by gradient xylene/alcohol and rehydrated by PBS. After that, hematoxylin and eosin staining were conducted on the tissue for 10 min and 1 min respectively. Masson staining procedure was according to the commercial kit for Masson staining (Servicebio, China) [29]. Finally, tissues were sealed with neutral resin and pictured by the microscope (ECHO Revolve, American).

### Immunofluorescence staining.

Dewaxed slices were blocked in 5% BSA and 0.5% Triton-X-100 (Servicebio, China) for 1 h at RT and were incubated with primary antibodies at 4℃ overnight. After washing for three times, tissues were incubated with corresponding second antibody for 1 h. Finally, the slices were observed and pictured.

For cell immunofluorescence staining, cells were seeded on glass slides and cultured. Corresponding treatments were conducted under the condition of serum-free DMEM. After washing with PBS, cells were fixed with 4% paraformaldehyde for 20 min. After being permeabilized, cells were incubated with primary antibodies: α-SMA (Abcam, ab7817) and Col 1 (Affinity, AF7001) at 4 ℃ overnight. Washed with TBST in triplicate, cells were incubated with secondary antibody for 60 min. Nuclei was stained with DAPI and the slices were pictured.

### Internalization measurements

NP was labeled with DIR (1,1-dioctadecy 1–3,3,3,3-tetramethy-lindotricarbocyanine iodide) (D12731, Invitrogen, Life Technologies) according to the manufacturer’s protocol. Then DIR-labeled Agomir-122@MNP, Agomir-122@M^m^NP, and Agomir-122@NP were obtained. Next, the three agents above was separately co-cultured with fibroblasts for 12 h, or with macrophages for 4 h. Culture medium was discarded afterwards and the dish was washed with PBS in triplicate. Cells were fixed in 4% PFA and stained with DAPI for 3 min. Finally, the fluoresecne of labeled agents internalized by fibroblasts was observed microscopically (ECHO Revolve, America), while that by macrophages was measured by flow cytometry.

### Tracing analysis

Labeled Agomir-122@M^m^NP was injected into the shoulder capsule of mice. The mice were subject to in vivo imaging at 1 day, 3 day, 5 day and 7 day post-injection by fluorescence imaging system (VISQUE InVivo ART, Wieworks) [22]. IVIS software (Living Image Software for IVIS) was used to observe the fluorescence intensity.

### Detection of apoptosis by Annexin V/Propidium iodide (PI)

The apoptosis rate of two fibroblasts was evaluated by Annexin V FITC and PI PE (C1062S, Beyotime) double staining according to the instructions [30]. In general, cells were collected, washed, and re-suspended in binding buffer. Then 5 μl fluorescein isothiocyanate-conjugated Annexin V and 10 μl PI were added, and incubated for 20 min in darkness. After that, the apoptosis rate was analyzed by flow cytometry.

### Bioinformatic analysis and target gene prediction

Heatmap diagram of the top 100 differentially expressed miRNAs as well as all differentially expressed miRNAs with statistical significance (n = 19) was drawn by Complex Heatmap R package. Biological pathway analysis was used to predict the relationship between miRNA and signal pathway according to an established manner [31].

### Statistical analysis

Data, as presented as mean ± SD, was analyzed using GraphPad Prism 7.0 (GraphPad Software, La Jolla, CA) and SPSS 18.0 software. Student-t test was used for comparisons of two groups. One-way analysis of variance (ANOVA) with post hoc test were applied to evaluate the differences between multiple groups. 2-tailed p < 0.05 was regarded as statistical significance.

### Supplementary Information

**Additional file 1: Figure S1. **The level of p-Smad2/3 relative to t-Smad2/3 in CDFs with different interventions. (n = 3). **: p < 0.01; ****p < 0.0001. **Figure S2. **Agomir-122@MNP inhibited TGF-β-induced activation of CDFs. **A** Cell proliferation of CDFs with different interventions, as determined by BrdU staining (green). **B** Quantification of BrdU staining. **C** Cell activity of CDFs with different interventions, as determined by CCK-8 assay. **D** Cell contraction ability of CDFs with different interventions, as determined by collagen gel contraction assay. **E** Quantification of collagen gel area. Scale bar: 200 μm. (n = 3). *p < 0.05; **p < 0.01; ***p < 0.001; ****p < 0.0001. **Figure S3. **Immunofluorescence of Col 1 (green) and α-SMA (red) expression on NIH3T3 cells (nuclei in DAPI blue) challenged with TGF-β (**A**), and quantification (**B**) (n = 3). *p < 0.05; **p < 0.01. **Figure S4. **Characterization of Agomir-122 loaded nanoparticles wrapped with cell membrane of activated NIH3T3 cells. **A** The level of α-SMA in NIH3T3 cells with TGF-β stimulation, determined by western blot, and quantification (n = 3). **B** SDS-PAGE analysis of NIH3T3 cells, Agomir-122M^m^NP, and Agomir-122@NP. **C** Morphology of Agmoir-122@NP and Agomir-122@M^m^NP, observed by STM. Scale bar: 100 nm. **D** Diameters of Agomir-122@NP and Agomir-122@M^m^NP (n = 3). **E** Zeta potentials of Agomir-122@NP and Agomir-122@M^m^NP (n = 3). **p < 0.01. I: NIH3T3, II: Agomir-122M^m^NP, III: Agomir-122@NP. **Figure S5. **Determining a concentration of Agomir-122 effective in up-regulating miR-122 in NIH3T3 cells (**A**), and the effectiveness of Agomir-122@NP and Agomir-122@M^m^NP in up-regulating miR-122 level, as compared to Agomir-122 alone, in NIH3T3 cells (**B**). (n = 3). **p < 0.01; ***p < 0.001. **Figure S6. **The biosafety of different agents on NIH3T3 cells, as measured by apoptosis flow cytometry. (n = 3). **Figure S7. **The uptake of Agomir-122@NP or Agomir-122@M^m^NP (DiR-labeled) by NIH3T3 cells stimulated or not stimulated with TGF-β, and quantification. (n = 3). Scale bar: 25 μm. ***p < 0.001. *NS* no significance. **Figure S8. **Immune evasion of Agomir-122@M^m^NP. Raw264.7 cells with internalized Agomir-122@NP or Agomir-122@M^m^NP were detected by flow cytometry, with quantification. (n = 3). ***p < 0.001; ****p < 0.0001. **Figure S9. **Agomir-122@M^m^NP inhibited TGF-β-induced activation of NIH3T3 cells. **A** Cell proliferation of NIH3T3 cells with different interventions, as determined by BrdU staining (green). **B** Quantification of BrdU staining. **C** Cell activity of NIH3T3 cells with different interventions, as determined by CCK-8 assay. **D** Cell contraction ability of NIH3T3 cells with different interventions, as determined by collagen gel contraction assay (red circle indicated the collagen gel). **E** Quantification of collagen gel area. Scale bar: 200 μm. (n = 3). I: NC, II: TGF-β, III: TGF-β + Agomir-122@NP, IV: TGF-β + Agomir-122@M^m^NP. *p < 0.05; **p < 0.01; ***p < 0.001; ****p < 0.0001. **Figure S10. **Evaluate the impact of Agomir-NC, NP, and MNP on activated NIH3T3 cells. **A** Assess intergroup cell proliferation capability through BrdU(green) staining. **B** Compare the vitality of NIH3T3 cells among different groups using CCK-8. **C** Statistical chart of BrdU-positive cells from BrdU staining. (n=3). Scale bar: 200 μm. *ns* no significance, *NC* Negative control. **Figure S11. **Representative live tracing image of agent injection into the index shoulder. One day, 3 days, 5 days, and 7 days after DiR-labeled Agomir-122@M^m^NP injection, fluorescent signaling was viewed at the injection site in a diminishing manner. **Figure S12. **Therapeutic effect of Agomir-122@M^m^NP against frozen shoulder in a mouse model. **A** Schematic chart of model establishment, intervention, as well as sample harvest and observation. **B** Representative histological image of mouse shoulder joint capsule with different interventions, as illustrated by HE and α-SMA staining (α-SMA in red and DAPI in blue). **C** Quantification of cell counts in joint capsule of different groups. **D** Quantification of joint capsule thickness of different groups. **E** α-SMA expression in joint capsule of different groups. **F** Passive ROM of the index shoulder of different groups. Scale bar: 200 or 100 μm. (n = 6). *p < 0.05; **p < 0.01; ***p < 0.001; ****p < 0.0001. **Figure S13. **HE staining of the main organs (heart, liver, spleen, lung, kidney and Intestine) after treatments. Scale bar: 100 μm.

## Data Availability

The data for this article can be found in the manuscript or in the Supplementary Material, which can be made available by the corresponding author upon reasonable request.
