# Supplementary material for: Agomir-122-loaded nanoparticles coated with cell membrane of activated fibroblasts to treat frozen shoulder based on homologous targeting
Source: J Nanobiotechnology. 2024 Apr 10;22:165. doi: 10.1186/s12951-024-02403-w (PMC11008019; doi:10.1186/s12951-024-02403-w)
Supplement: Supplementary file 1 — Additional file 1: Figure S1. The level of p-Smad2/3 relative to t-Smad2/3 in CDFs with different interventions. (n = 3). **: p < 0.01; ****p < 0.0001. Figure S2. Agomir-122@MNP inhibited TGF-β-induced activation of CDFs. A Cell proliferation of CDFs with different interventions, as determined by BrdU staining (green). B Quantification of BrdU staining. C Cell activity of CDFs with different interventions, as determined by CCK-8 assay. D Cell contraction ability of CDFs with different interventions, as determined by collagen gel contraction assay. E Quantification of collagen gel area. Scale bar: 200 μm. (n = 3). *p < 0.05; **p < 0.01; ***p < 0.001; ****p < 0.0001. Figure S3. Immunofluorescence of Col 1 (green) and α-SMA (red) expression on NIH3T3 cells (nuclei in DAPI blue) challenged with TGF-β (A), and quantification (B) (n = 3). *p < 0.05; **p < 0.01. Figure S4. Characterization of Agomir-122 loaded nanoparticles wrapped with cell membrane of activated NIH3T3 cells. A The level of α-SMA in NIH3T3 cells with TGF-β stimulation, determined by western blot, and quantification (n = 3). B SDS-PAGE analysis of NIH3T3 cells, Agomir-122MmNP, and Agomir-122@NP. C Morphology of Agmoir-122@NP and Agomir-122@MmNP, observed by STM. Scale bar: 100 nm. D Diameters of Agomir-122@NP and Agomir-122@MmNP (n = 3). E Zeta potentials of Agomir-122@NP and Agomir-122@MmNP (n = 3). **p < 0.01. I: NIH3T3, II: Agomir-122MmNP, III: Agomir-122@NP. Figure S5. Determining a concentration of Agomir-122 effective in up-regulating miR-122 in NIH3T3 cells (A), and the effectiveness of Agomir-122@NP and Agomir-122@MmNP in up-regulating miR-122 level, as compared to Agomir-122 alone, in NIH3T3 cells (B). (n = 3). **p < 0.01; ***p < 0.001. Figure S6. The biosafety of different agents on NIH3T3 cells, as measured by apoptosis flow cytometry. (n = 3). Figure S7. The uptake of Agomir-122@NP or Agomir-122@MmNP (DiR-labeled) by NIH3T3 cells stimulated or not stimulated with TGF-β, and quantification. (n = [file 12951_2024_2403_MOESM1_ESM.docx]

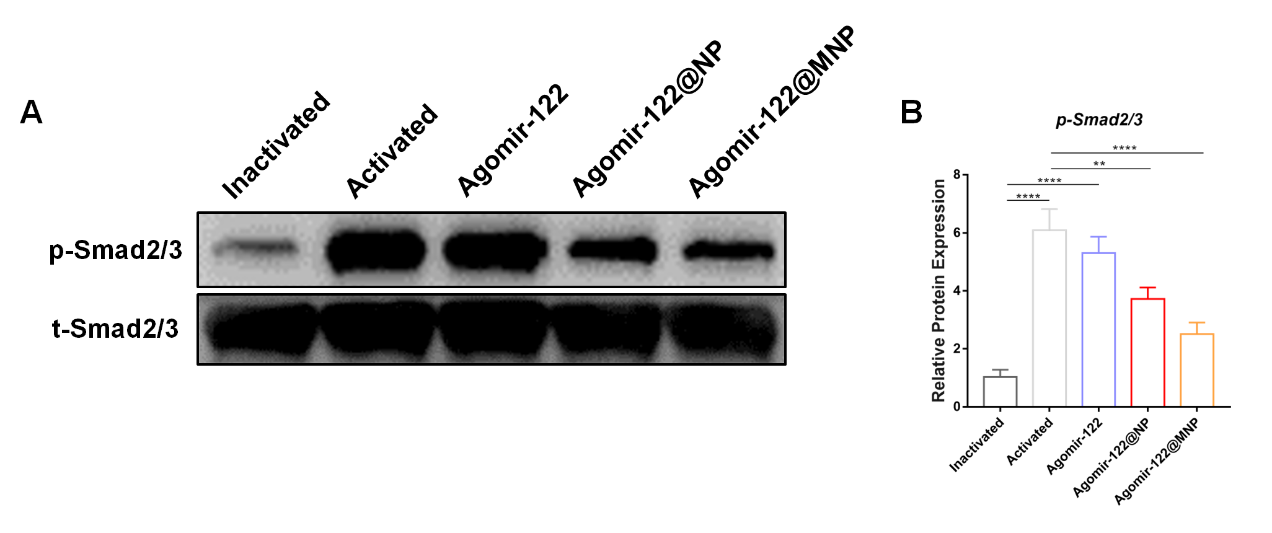


Figure S1.

The level of p-Smad2/3 relative to t-Smad2/3 in CDFs with different interventions. (n = 3). **: p < 0.01; ****: p < 0.0001.


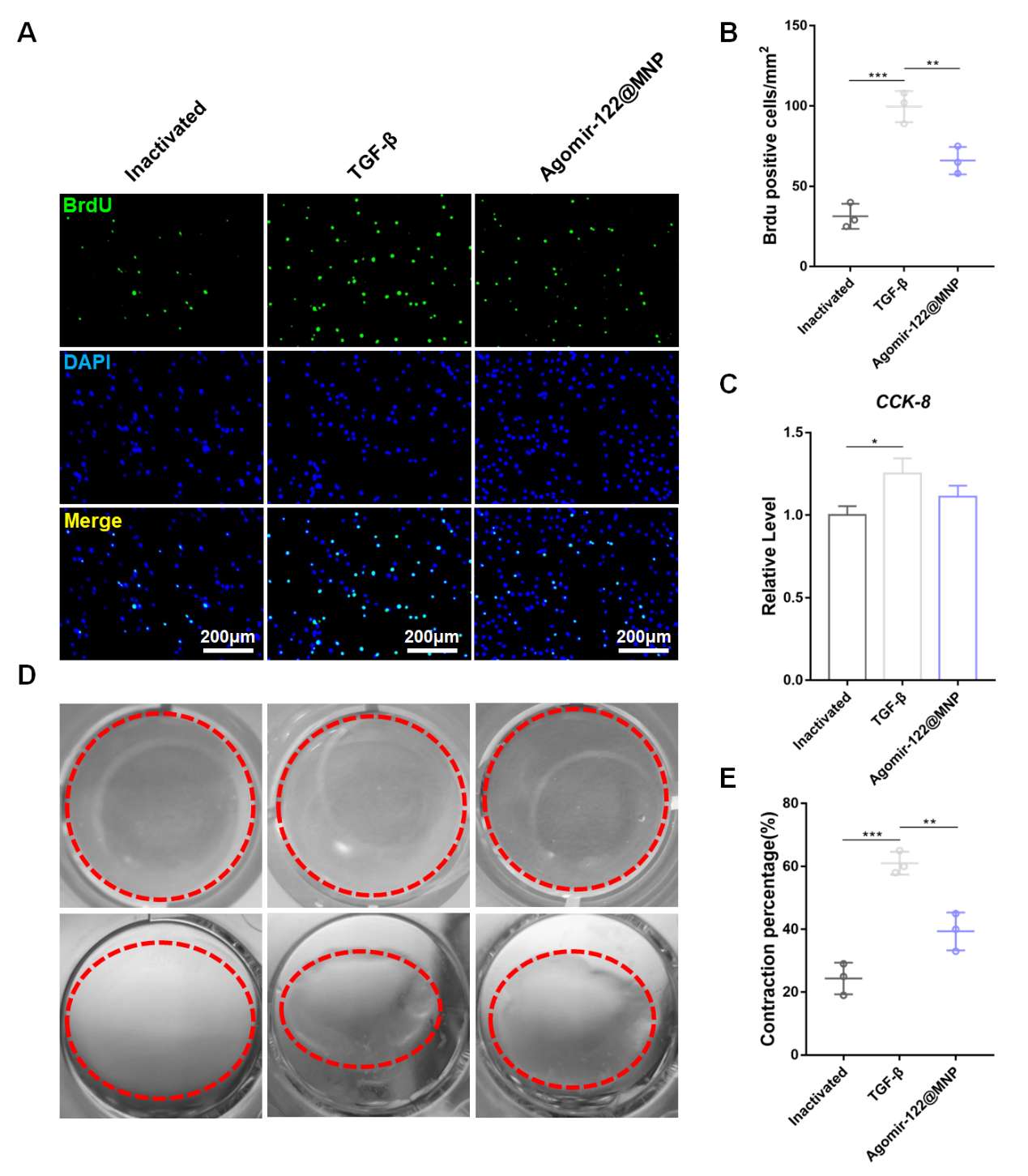


Figure S2.

Agomir-122@MNP inhibited TGF-β-induced activation of CDFs. A) Cell proliferation of CDFs with different interventions, as determined by BrdU staining (green). B) Quantification of BrdU staining. C) Cell activity of CDFs with different interventions, as determined by CCK-8 assay. D) Cell contraction ability of CDFs with different interventions, as determined by collagen gel contraction assay. E) Quantification of collagen gel area. Scale bar: 200 μm. (n = 3). *: p < 0.05; **: p < 0.01; ***: p < 0.001; ****: p < 0.0001.


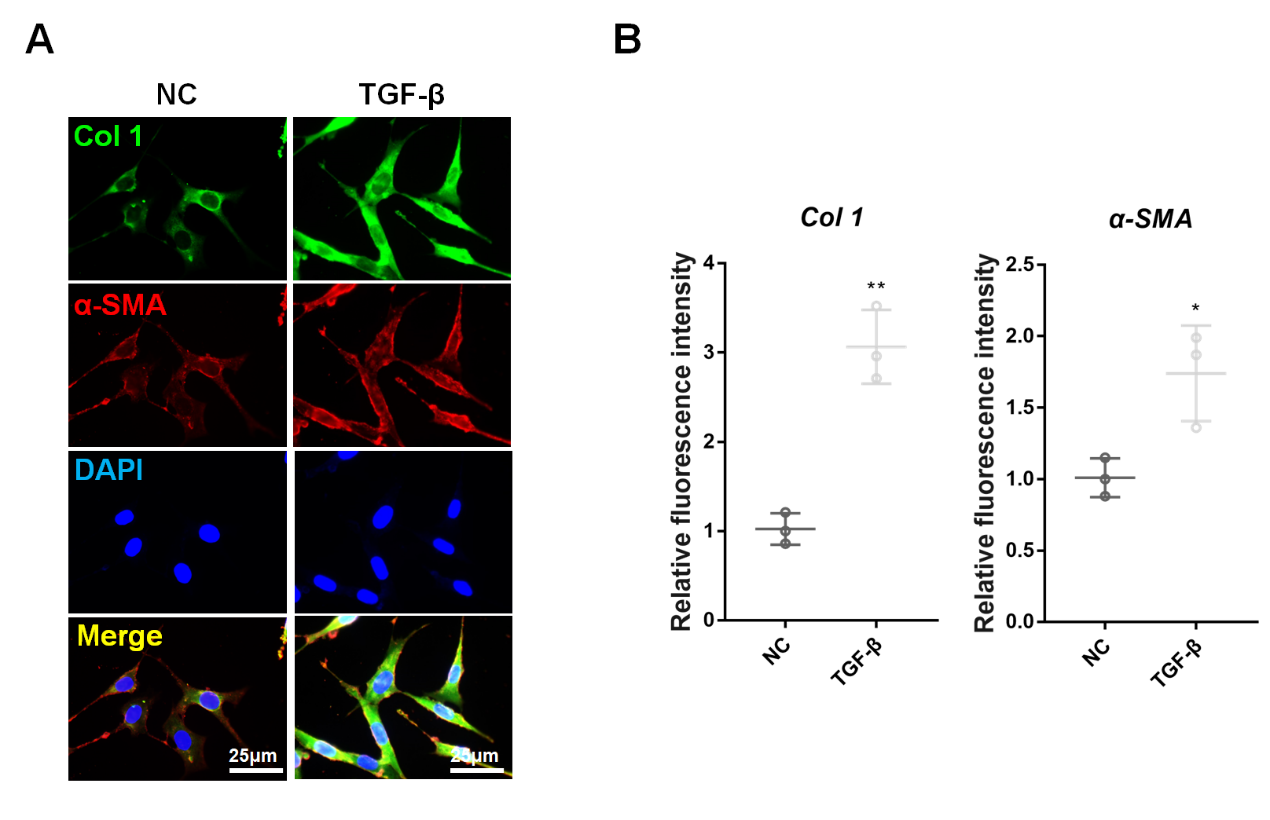


Figure S3.

Immunofluorescence of Col 1 (green) and α-SMA (red) expression on NIH3T3 cells (nuclei in DAPI blue) challenged with TGF-β (A), and quantification (B) (n = 3). *: p < 0.05; **: p < 0.01.


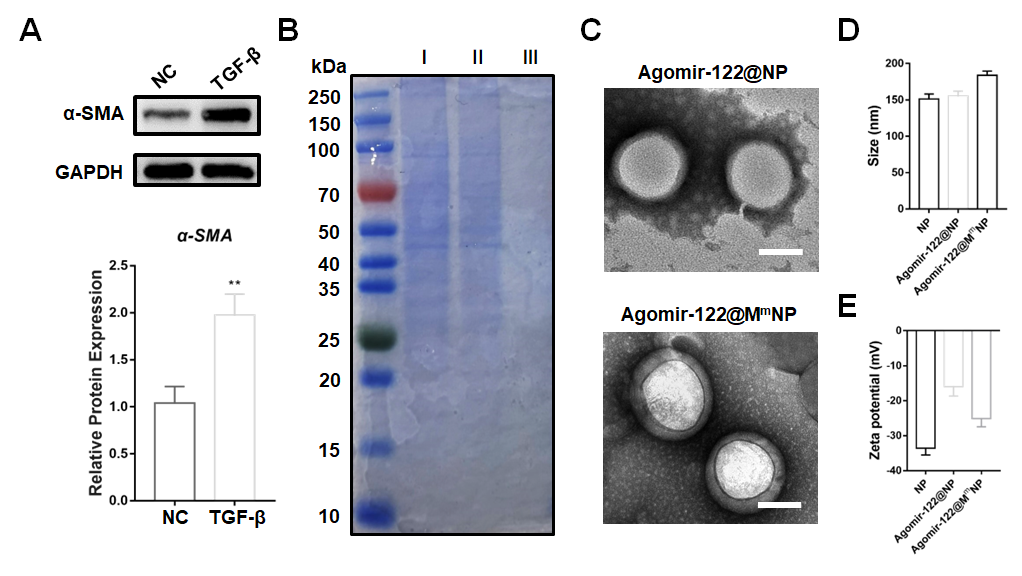


Figure S4.

Characterization of Agomir-122 loaded nanoparticles wrapped with cell membrane of activated NIH3T3 cells. A) The level of α-SMA in NIH3T3 cells with TGF-β stimulation, determined by western blot, and quantification (n = 3). B) SDS-PAGE analysis of NIH3T3 cells, Agomir-122M^m^NP, and Agomir-122@NP. C) Morphology of Agmoir-122@NP and Agomir-122@M^m^NP, observed by STM. Scale bar: 100 nm. D) Diameters of Agomir-122@NP and Agomir-122@M^m^NP (n = 3). E) Zeta potentials of Agomir-122@NP and Agomir-122@M^m^NP (n = 3). **: p < 0.01. Ⅰ: NIH3T3, Ⅱ: Agomir-122M^m^NP, Ⅲ: Agomir-122@NP.


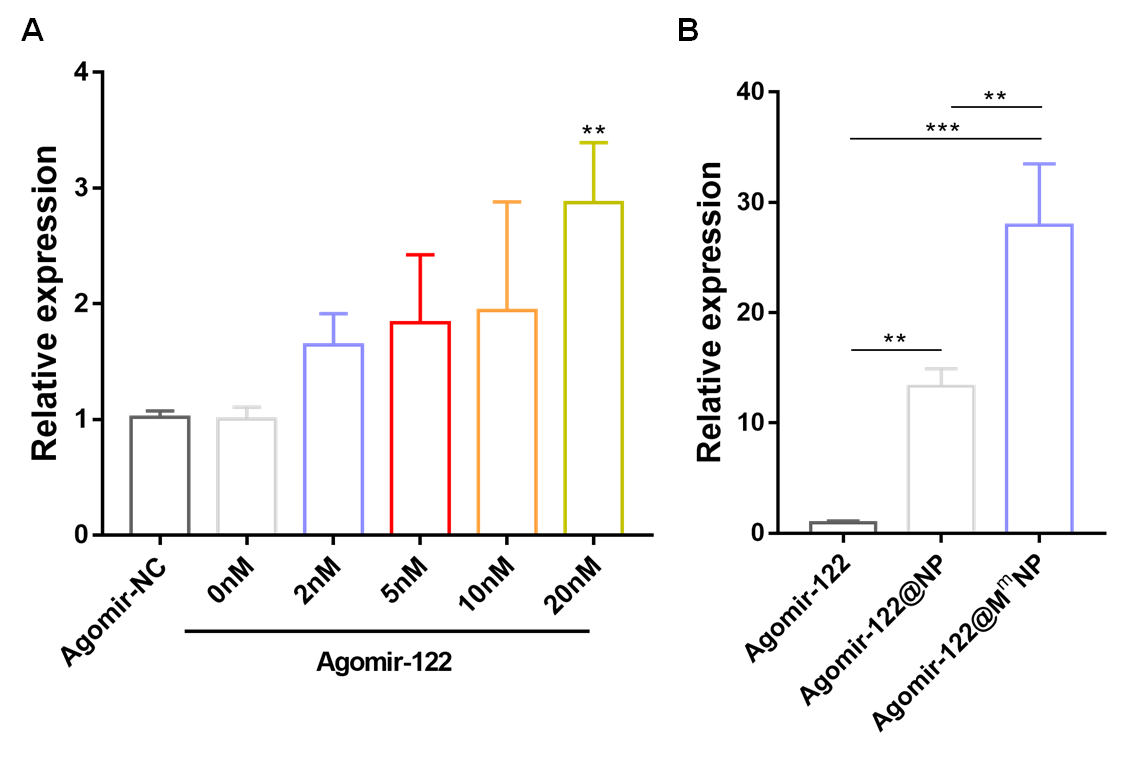


Figure S5.

Determining a concentration of Agomir-122 effective in up-regulating miR-122 in NIH3T3 cells (A), and the effectiveness of Agomir-122@NP and Agomir-122@M^m^NP in up-regulating miR-122 level, as compared to Agomir-122 alone, in NIH3T3 cells (B). (n = 3). **: p < 0.01; ***: p < 0.001.


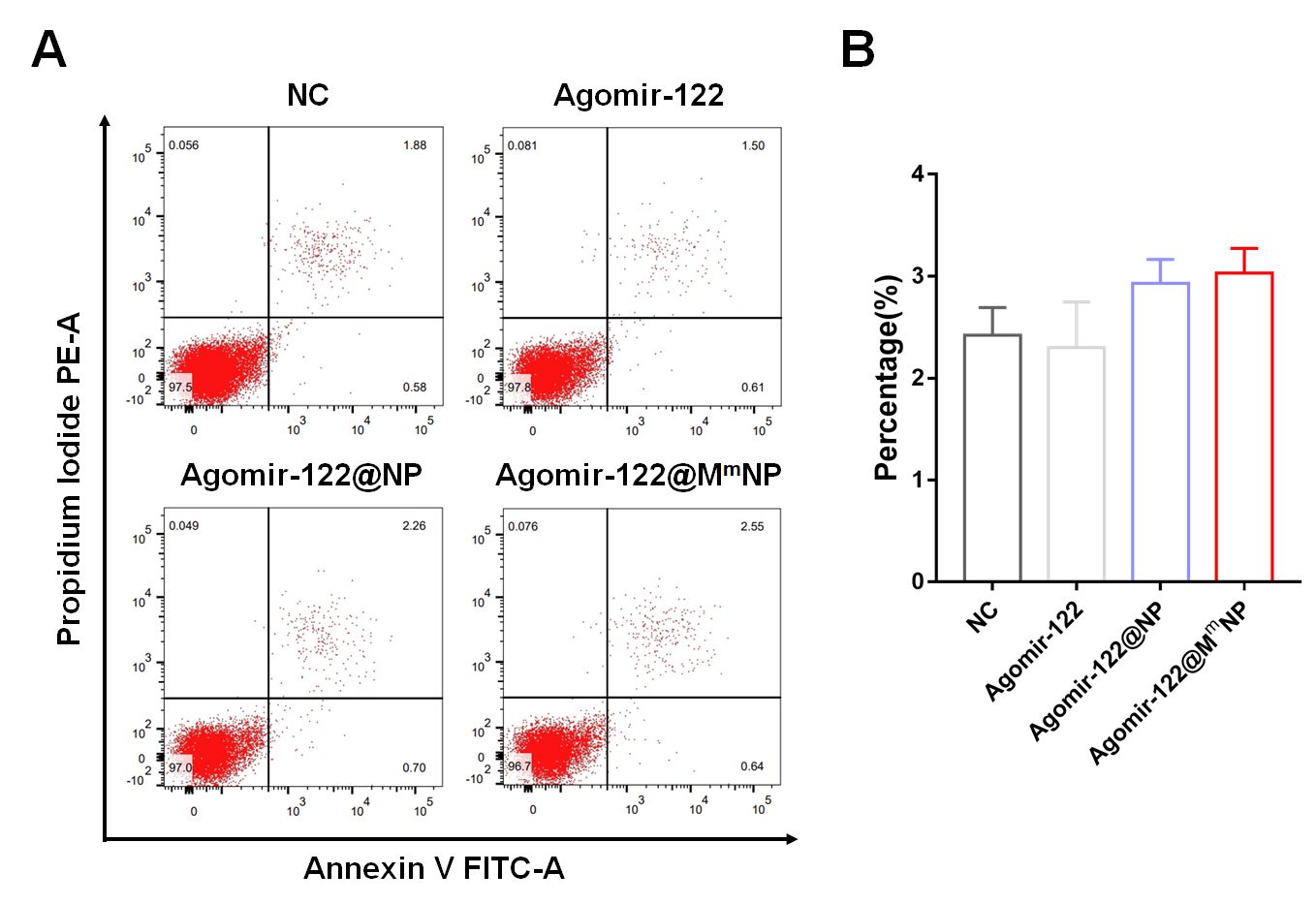


Figure S6.

The biosafety of different agents on NIH3T3 cells, as measured by apoptosis flow cytometry. (n = 3).


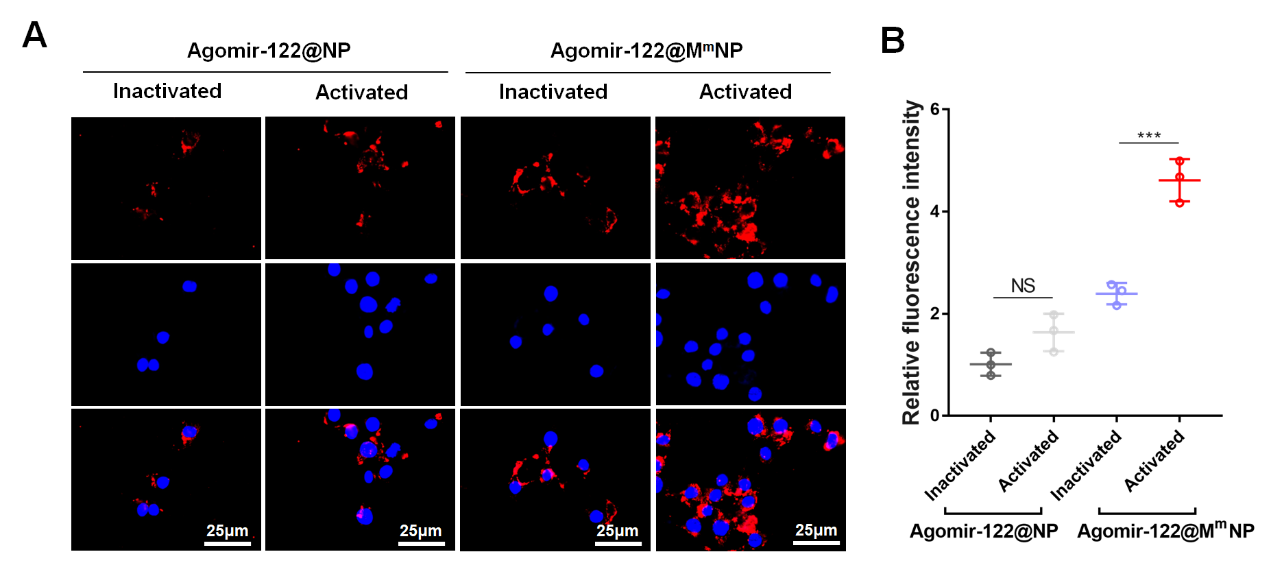


Figure S7.

The uptake of Agomir-122@NP or Agomir-122@M^m^NP (DiR-labeled) by NIH3T3 cells stimulated or not stimulated with TGF-β, and quantification. (n = 3). Scale bar: 25 μm. ***: p < 0.001. NS: no significance.


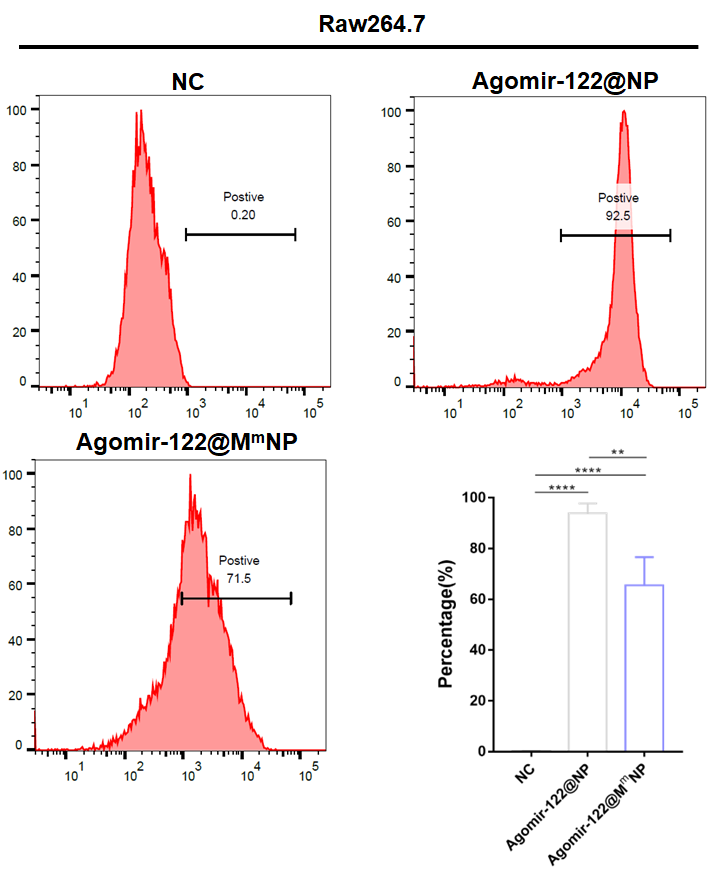


Figure S8.

Immune evasion of Agomir-122@M^m^NP. Raw264.7 cells with internalized Agomir-122@NP or Agomir-122@M^m^NP were detected by flow cytometry, with quantification. (n = 3). ***: p < 0.001; ****: p < 0.0001.


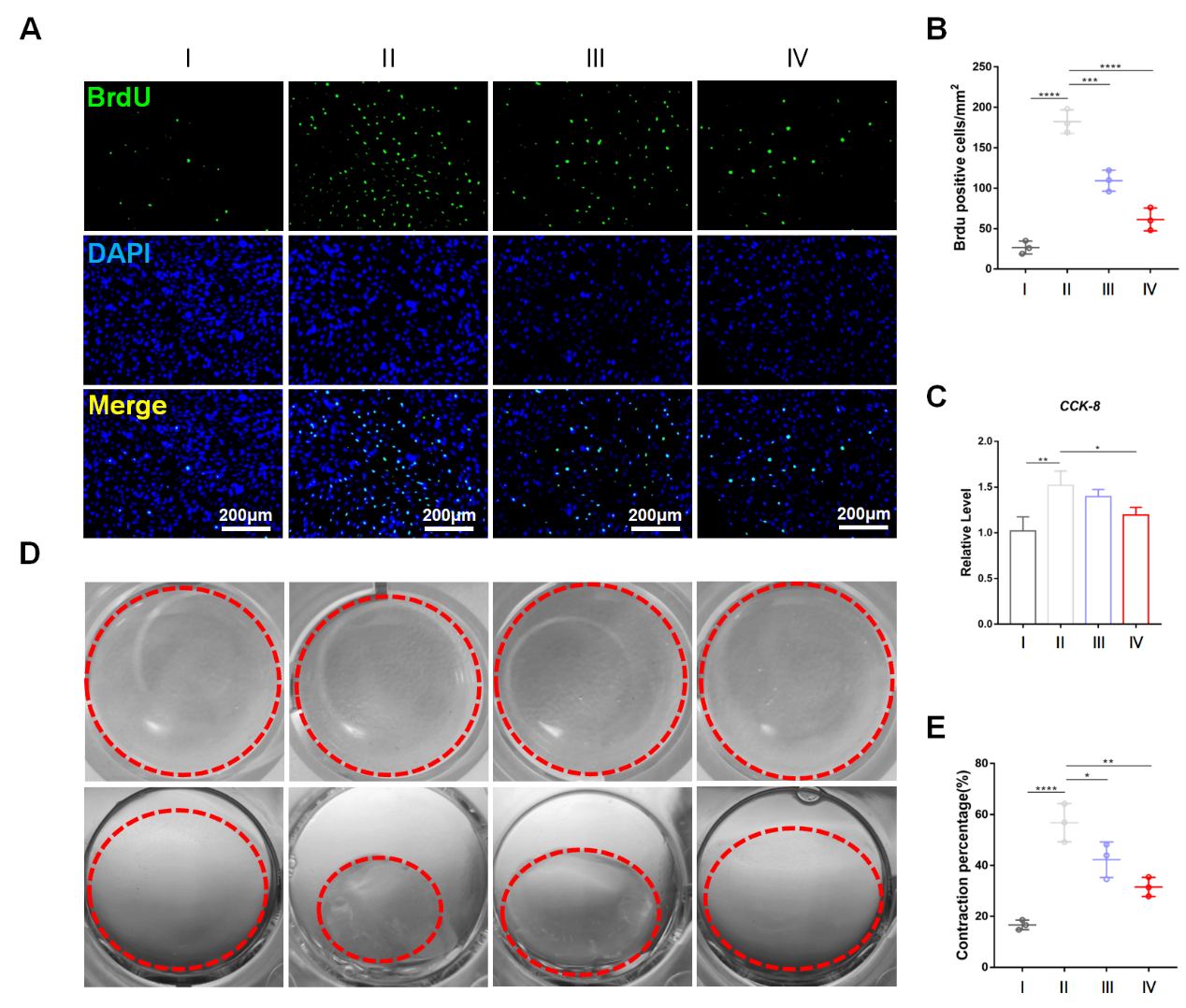


Figure S9.

Agomir-122@M^m^NP inhibited TGF-β-induced activation of NIH3T3 cells. A) Cell proliferation of NIH3T3 cells with different interventions, as determined by BrdU staining (green). B) Quantification of BrdU staining. C) Cell activity of NIH3T3 cells with different interventions, as determined by CCK-8 assay. D) Cell contraction ability of NIH3T3 cells with different interventions, as determined by collagen gel contraction assay (red circle indicated the collagen gel). E) Quantification of collagen gel area. Scale bar: 200 μm. (n = 3). I: NC, Ⅱ: TGF-β, III: TGF-β + Agomir-122@NP, IV: TGF-β + Agomir-122@M^m^NP. *: p < 0.05; **: p < 0.01; ***: p < 0.001; ****: p < 0.0001.


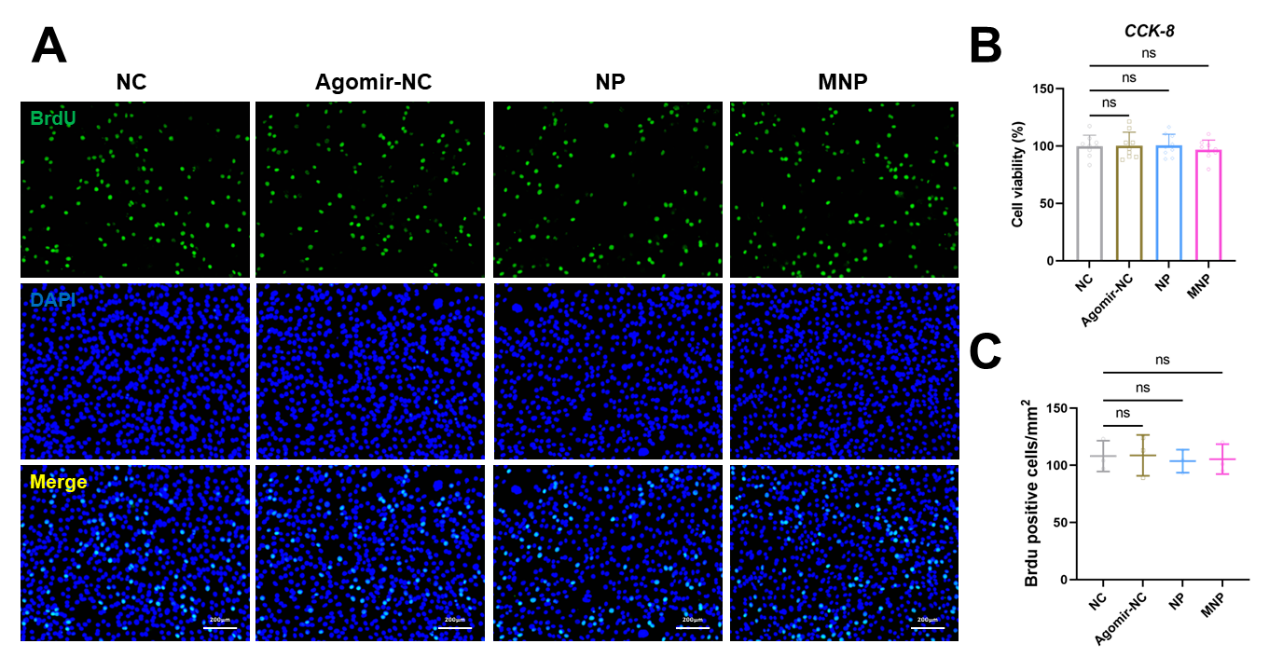


Figure S10.

Evaluate the impact of Agomir-NC, NP, and MNP on activated NIH3T3 cells. A) Assess intergroup cell proliferation capability through BrdU(green) staining. B) Compare the vitality of NIH3T3 cells among different groups using CCK-8. C) Statistical chart of BrdU-positive cells from BrdU staining. (n=3). Scale bar: 200 μm. ns: no significance. NC: Negative control.


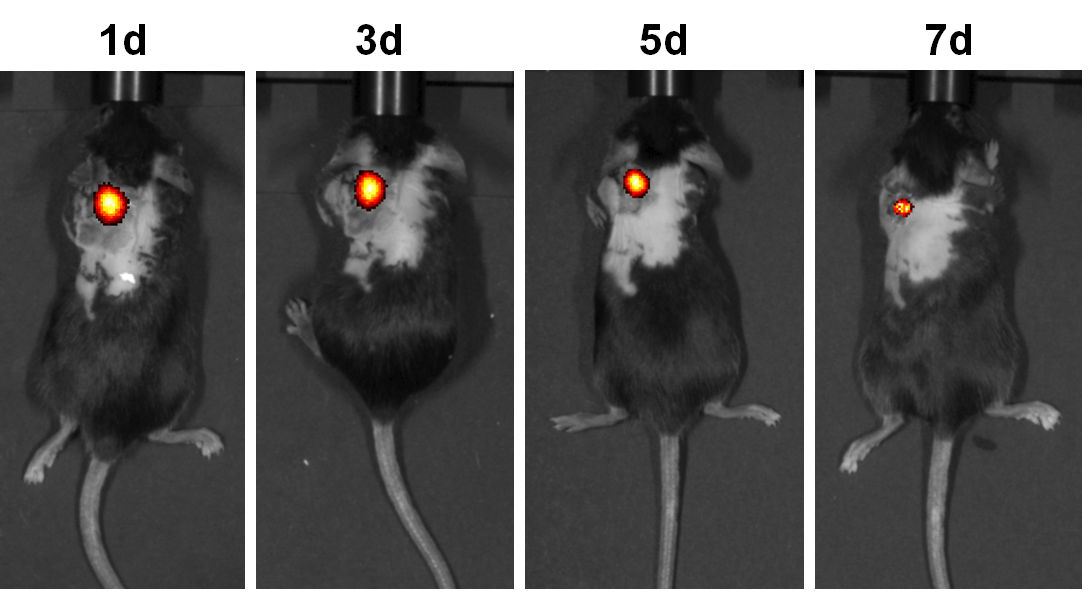


Figure S11.

Representative live tracing image of agent injection into the index shoulder. One day, three days, five days, and seven days after DiR-labeled Agomir-122@M^m^NP injection, fluorescent signaling was viewed at the injection site in a diminishing manner.


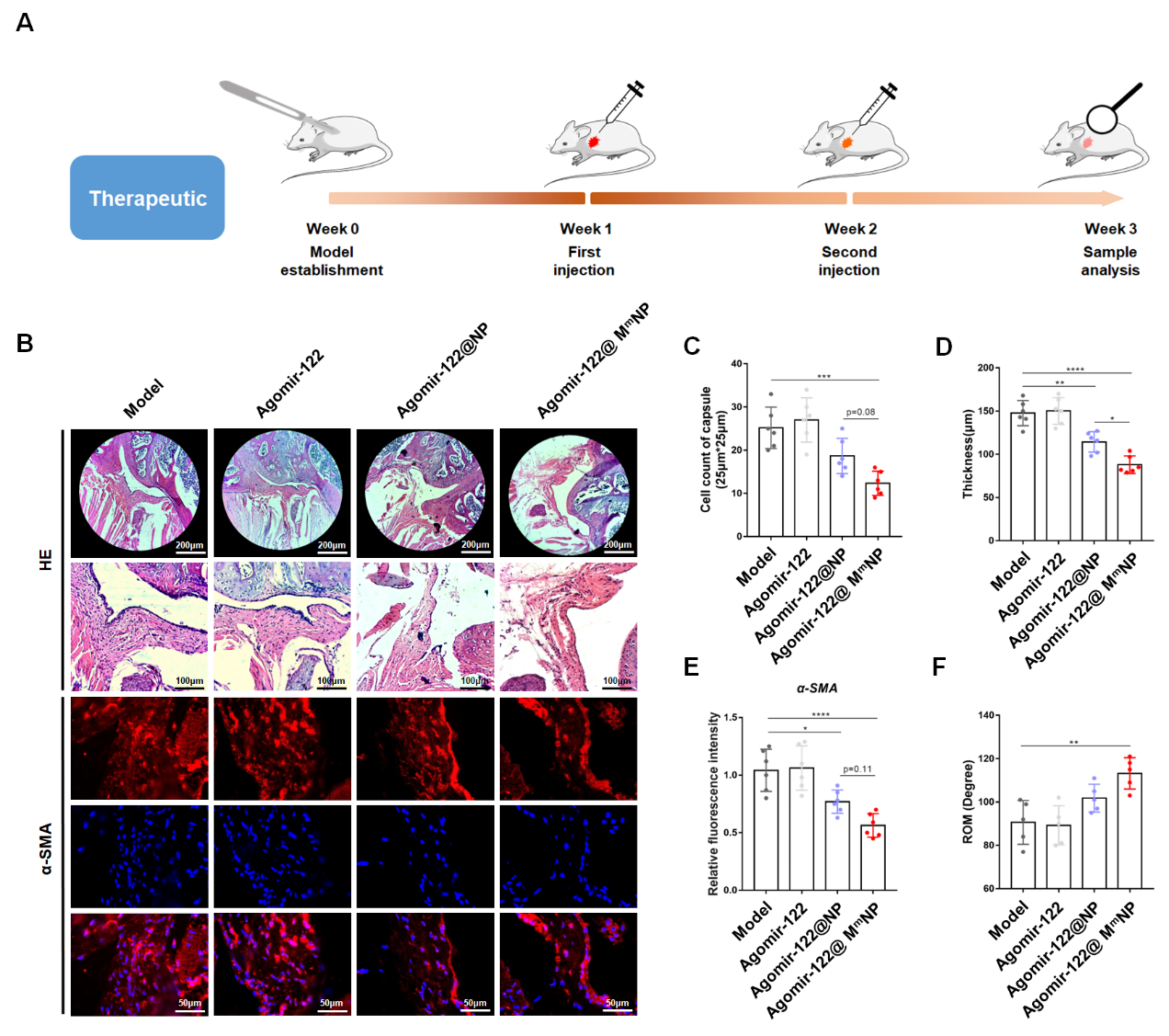


Figure S12.

Therapeutic effect of Agomir-122@M^m^NP against frozen shoulder in a mouse model. A) Schematic chart of model establishment, intervention, as well as sample harvest and observation. B) Representative histological image of mouse shoulder joint capsule with different interventions, as illustrated by HE and α-SMA staining (α-SMA in red and DAPI in blue). C) Quantification of cell counts in joint capsule of different groups. D) Quantification of joint capsule thickness of different groups. E) α-SMA expression in joint capsule of different groups. F) Passive ROM of the index shoulder of different groups. Scale bar: 200 or 100 μm. (n = 6). *: p < 0.05; **: p < 0.01; ***: p < 0.001; ****: p < 0.0001.


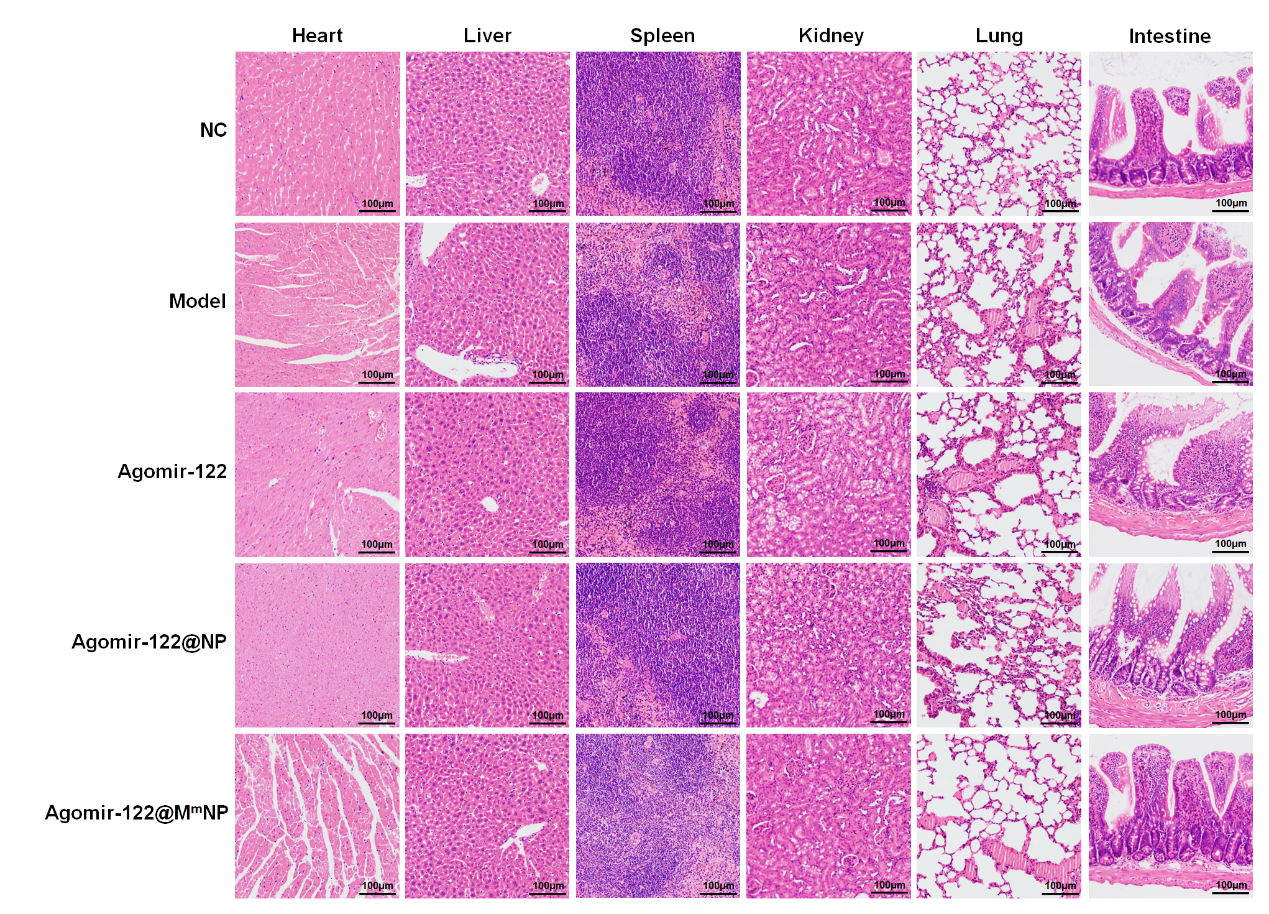


Figure S13.

HE staining of the main organs (heart, liver, spleen, lung, kidney and Intestine) after treatments. Scale bar: 100 μm
